# Gender Differences in Well–Being Among People Age ≥ 50 in Medication-Assisted Treatment for Opioid Use Disorder

**DOI:** 10.1093/geroni/igab046.1116

**Published:** 2021-12-17

**Authors:** Sahil Angelo, Mary Mitchell, Molly Perkins, Alexis Bender

**Affiliations:** 1 Medstar Georgetown University Hospital, Medstar Georgetown University Hospital, District of Columbia, United States; 2 Friends Research Institute, Baltimore, Maryland, United States; 3 Emory University of Medicine, Atlanta, Georgia, United States; 4 Emory University, Atlanta, Georgia, United States

## Abstract

The impact of Adverse Childhood Experiences (ACEs) on the physical and mental well–being of older adults with Opioid Use Disorder (OUD) is unclear, and we know even less about gender differences. This study explores this association and investigates additional factors (e.g., pain, depression) that may affect physical and mental well–being in this population with a focus on gender. The sample for the present analysis includes 90 adults aged 50 and older from a larger study focused on aging with OUD across eight opioid treatment programs in Georgia. We performed multivariable linear regression analyses by gender. There was a small, but significant, association between ACEs and mental well–being for men only. Other significant predictors of physical and mental well-being (e.g., insurance status, pain, satisfaction with social role, stigma) varied by gender. We discuss the importance of these gender differences in identifying appropriate areas for intervention to improve physical and mental well–being.

